# Myeloid EGR1 contributes to Th17/Treg imbalance during acute liver allograft rejection in a rat model

**DOI:** 10.3389/fimmu.2026.1901189

**Published:** 2026-09-21

**Authors:** Yaming Ding, Jie Wang, Shulin Li, Guoxiang Shen, Lihong Chen

**Affiliations:** 1 Department of Pathology and Institute of Oncology, The School of Basic Medical Sciences, Fujian Medical University, Fuzhou, Fujian, China; 2 Diagnostic Pathology Center, Fujian Medical University, Fuzhou, Fujian, China

**Keywords:** acute rejection (AR), early growth response protein 1 (EGR1), liver transplantation, myeloid cells, single-cell RNA sequencing, Th17/Treg balance

## Abstract

**Background:**

Ischemia-reperfusion injury (IRI) and acute rejection (AR) limit liver transplantation success. Early growth response 1 (EGR1) mediates stress and inflammation, yet its cellular distribution and potential involvement in adaptive alloimmunity during distinct post-transplant stages remain unclear.

**Methods:**

A rat orthotopic liver transplantation model was used to evaluate graft injury and EGR1 spatiotemporal expression during IRI (6–24 h) and AR (5–10 d). Public scRNA-seq data and virtual knockout analysis were used to assess the cellular distribution and potential regulatory role of *Egr1*. EGR1-overexpressing bone marrow-derived dendritic cells (BMDCs) were co-cultured with CD4^+^ T-cells to assess cytokine secretion, proliferation, and shifted Th17/Treg differentiation.

**Results:**

Both syngeneic and allogeneic grafts exhibited early IRI. By days 5–10, allografts developed AR with elevated liver enzymes and Banff scores. EGR1 rose transiently during IRI but re-escalated exclusively in allografts during AR, localizing to portal infiltrates. scRNA-seq showed preferential *Egr1* expression in graft myeloid cells, while virtual knockout suggested immune-related regulatory effects. EGR1-associated APC activation and Th17-associated responses were observed in rejecting allografts. EGR1 overexpression in BMDCs promoted pro-inflammatory cytokine secretion, T-cell proliferation, and altered Th17/Treg-associated responses.

**Conclusion:**

EGR1 contributes to liver allograft rejection by modulating myeloid APC responses and Th17/Treg-associated T-cell responses, highlighting its potential as a therapeutic target for AR.

## 1 Introduction

Liver transplantation remains the definitive treatment for end-stage liver diseases (1, 2). Despite continuous improvements in surgical techniques and immunosuppressive regimens, postoperative ischemia-reperfusion injury (IRI) and acute rejection (AR) remain critical obstacles to long-term graft survival (3, 4). IRI typically occurs during the early postoperative phase (6–24 hours) and is primarily driven by sterile inflammation (5). In7nbsp;contrast, AR usually manifests between 5 to 10 days post-transplantation and is predominantly mediated by alloreactive T cells. During both IRI and AR, the robust activation of immune cells and the subsequent release of inflammatory cytokines contribute significantly to postoperative liver injury (6). Although current immunosuppressive drugs effectively mitigate immune activation, their non-specific inhibition causes severe adverse effects, including an increased risk of infections, *de novo* malignancies, and dysmetabolic syndromes (7). Therefore, there is an urgent need to develop novel, highly specific therapeutic strategies to modulate the immune system, thereby improving the long-term quality of life and prolonging the viability of liver allografts.

Various immune mediators that trigger inflammatory cascades are associated with cellular responses during IRI or AR in liver transplantation. These mediators represent potential therapeutic targets for mitigating excessive post-transplant immune activation (8, 9). Early growth response 1 (EGR1) is an immediate-early transcription factor that rapidly responds to stimuli such as hypoxia, inflammatory mediators, and tissue damage. By upregulating the transcription of multiple proinflammatory genes, including interleukin-1β (IL-1β) and tumor necrosis factor-alpha (TNF-α), EGR1 functions as a master switch regulator for inflammatory cascades (10, 11). Previous studies using cardiac, pulmonary, and tracheal transplant models have demonstrated that *Egr1* deficiency or downregulation is associated with decreased expression of inflammatory mediators, attenuated immune cell infiltration, and alleviated allograft rejection (10, 12, 13). However, these studies primarily evaluated global EGR1 expression. Its specific cellular sources and dynamic functional changes across distinct phases of liver transplantation remain poorly defined. Because IRI and AR occur sequentially but are driven by distinct mechanisms, conventional bulk tissue analyses fail to discriminate between early ischemic stress and subsequent adaptive alloimmune activation. Furthermore, while T cells act as the ultimate effectors of AR, their activation is fundamentally dependent on upstream myeloid antigen-presenting cells (APCs) (14). Crucially, the specific role of EGR1 within these myeloid APCs and its subsequent impact on downstream T-cell differentiation remain unexplored (15). Moreover, because EGR1 is an immediate-early and stress-responsive transcription factor, determining its cellular distribution and functional context at single-cell resolution is particularly important for distinguishing biologically relevant activation from generalized tissue-level changes.

In this study, we investigated the role of EGR1 during liver transplantation, with particular focus on its involvement in myeloid immune responses and subsequent T-cell regulation. We hypothesized that EGR1 is reactivated during AR and that EGR1 in graft myeloid cells modulates inflammatory responses and downstream CD4^+^ T-cell responses. Using a rat orthotopic liver transplantation model combined with single-cell RNA sequencing (scRNA-seq), histological analyses, and *in vitro* functional assays, we characterized the temporal expression of EGR1 across IRI and AR and its cellular distribution during AR. We further evaluated whether EGR1 expression in bone marrow-derived dendritic cells affects inflammatory cytokine production and CD4^+^ T-cell responses. Together, these findings suggest that myeloid EGR1 may contribute to immune regulation during liver allograft rejection.

## 2 Materials and methods

### 2.1 Animals and ethical statement

Male Lewis and Brown Norway (BN) rats (weighing 250–300 g) were purchased from the Laboratory Animal Center of Fujian Medical University, China (Permission No. SCXK(Min)2012-0001). All rats were housed in specific pathogen-free conditions with a controlled temperature (22 ± 2 °C) and humidity (50 ± 10%) under a 12-h light/12-h dark cycle, with ad libitum access to food and water. Animal experiments were conducted in accordance with the NIH Guidelines for the Care and Use of Laboratory Animals. The protocols used in this study were approved by the Institutional Animal Care and Use Committee (IACUC) of Fujian Medical University (Approval No. IACUC FJMU 2024-Y-0540).

### 2.2 Transplant techniques

Rats were allocated into the syngraft group (Lewis → Lewis), allograft group (Lewis → BN), and a sham group (Lewis only). The rat orthotopic liver transplantation (ROLT) model was established as previously described (16, 17). Each recipient rat was considered an independent biological replicate. Liver tissues and serum were collected from successfully modeled recipient rats at 6 h, 12 h, 24 h, 5 d, and 10 d after transplantation (n = 3 per group at each time point) for downstream analyses.

### 2.3 Histopathological evaluation and liver function detection

The graft liver was dissected according to its anatomical lobes, with tissues from each lobe collected and preserved. Samples from the left accessory lobe were preferentially used for histological and molecular analyses. Harvested tissues were fixed in 10% formaldehyde, embedded in paraffin, sectioned (5 µm), and stained with hematoxylin and eosin (H&E). Grading of ischemia-reperfusion injury was performed according to Suzuki’s criteria, and the rejection activity index was assessed according to the Banff criteria (18, 19).

Approximately 5 mL of blood was collected from the inferior vena cava under anesthesia and centrifuged to separate the serum. The serum levels of AST and ALT were detected by a commercial kit according to the manufacturer’s instructions (C010-2, C009-2, Nanjing Jiancheng, Nanjing, China).

### 2.4 Enzyme-linked immunosorbent assay

Blood samples were collected as described above. Serum levels of EGR1, IL-10, IFN-γ, and IL-1β in the sera of recipient rats, and the levels of IL-6, IL-12, TNF-α, and IL-10 in bone marrow-derived dendritic cell (BMDC) culture supernatants, were measured by ELISA according to the manufacturer’s instructions.

### 2.5 RNA isolation and real-time quantitative PCR

Total RNA from grafted livers was extracted using the RNAprep Pure Tissue Kit (Tiangen, China) and reverse-transcribed into cDNA utilizing the PrimeScript 1st Strand cDNA Synthesis Kit (Takara, Kusatsu, Japan). Amplification reactions were performed in triplicate on an ABI 7500 Real-Time PCR System (ABI, Life Technologies, USA) with the QuantiNova™ SYBR Green PCR Kit (Qiagen, Germany). The relative mRNA expression levels of target genes (*Egr1*, *Il6*, and *Il10*) were calculated using the 2−ΔΔCt method and normalized to *18S rRNA*. Fold changes in the syngraft and allograft groups were calculated relative to the sham group at 6 h post-transplantation. All primer sequences are detailed in **Supplementary Table S1** (SDC, Materials and Methods).

### 2.6 Western blot assay

Total protein from hepatic tissues and cultured BMDCs was extracted using RIPA lysis buffer, and protein concentrations were determined using a BCA Protein Assay Kit. Equal amounts of protein were separated by SDS-PAGE and transferred onto nitrocellulose membranes. After blocking, the membranes were incubated with primary antibodies against EGR1 (1:10,000, Novus Biologicals) and GAPDH (1:10,000, Sigma, USA), followed by peroxidase-conjugated secondary antibodies. Protein bands were visualized using an ECL substrate, and densitometry quantification was performed utilizing ImageJ software (National Institutes of Health, Bethesda, MD).

### 2.7 Immunohistochemistry

Grafted liver sections were subjected to antigen retrieval in citrate buffer (pH 6.0) and endogenous peroxidase blocking with 3% H_2_O_2_. After blocking with 1% BSA, sections were incubated with primary antibodies against EGR1 (1:250, ab55160, Abcam, UK), IL-17A (1:1000, ab318150, Abcam, UK), or CD86 (1:100, CY5238, Abways, China), followed by an HRP-conjugated secondary antibody (1:250, Abcam, UK). Immunoreactivity was visualized using DAB, and nuclei were counterstained with hematoxylin. EGR1-positive cells were quantified as positive cells per field, whereas CD86- and IL-17A-positive staining was quantified as the percentage of positive area using ImageJ. For each animal, measurements from multiple fields were averaged to obtain one biological replicate for statistical analysis.

### 2.8 Single-cell RNA sequencing data processing and cell-type annotation

Single-cell RNA sequencing data from rat liver grafts were obtained from the publicly available Gene Expression Omnibus dataset GSE285476 (20). For the present analysis, liver samples collected on day 7 after transplantation from the syngeneic and rejection groups were included, with three biological replicates in each group. The gene-expression matrices were imported into R and analyzed using the Seurat package.

Quality control was performed separately for individual samples before data integration. Cells were retained when they met all of the following criteria: 300 < nFeature_RNA < 5,500, 500 < nCount_RNA < 35,000, mitochondrial transcript proportion < 25%, and hemoglobin-gene transcript proportion < 1%. Cells not satisfying these criteria were excluded from downstream analyses. Following quality-control filtering, data were normalized using SCTransform. Principal component analysis was performed, followed by Harmony-based batch correction using individual sample identity as the batch variable.

The expression matrix was normalized and scaled, and highly variable genes were identified for dimensionality reduction. Principal component analysis (PCA) was subsequently performed, and the first 40 principal components (PCs 1–40) were used for neighborhood graph construction and UMAP visualization. Unsupervised clustering was performed using a graph-based clustering algorithm with a resolution parameter of 0.4.

Cell identities were assigned through a combination of reference-based annotation and manual marker-gene assessment. Preliminary cell-type annotation was performed using SingleR. Cluster-specific marker genes were subsequently identified using Seurat differential-expression analysis, and the top-ranked marker genes of each cluster were examined together with canonical lineage markers reported in previous single-cell studies of the liver and liver transplantation. Final annotations were determined by integrating the SingleR predictions, expression patterns of established marker genes, and cluster-specific differential-expression profiles. Major cell populations identified in the dataset included hepatocytes, cholangiocytes, endothelial cells, hepatic stellate cells, fibroblasts, myeloid cells, and lymphoid cells.

For comparisons between transplantation conditions, cells were stratified according to the original biological sample and experimental group. The cellular distribution and expression of *Egr1* and other stress-responsive genes were visualized using UMAP feature plots and dot plots. Cell-type-specific expression patterns were further examined to characterize the relative enrichment of *Egr1* across the major cellular compartments in syngeneic and rejecting liver grafts.

### 2.9 Virtual knockout analysis using scTenifoldKnk

To investigate the potential regulatory role of Egr1 in graft myeloid cells, the annotated myeloid-cell population was extracted from the single-cell dataset and subjected to virtual knockout analysis using the scTenifoldKnk R package (21). The gene-by-cell expression matrix of myeloid cells was used to construct a single-cell gene regulatory network (scGRN). A pseudo-knockout network was subsequently generated by virtually removing the regulatory influence of Egr1. The original and pseudo-knockout networks were compared through manifold alignment, and genes exhibiting significant changes in network regulation were identified as differentially regulated (DR) genes, herein referred to as virtual-KO perturbed genes.

The magnitude of network perturbation for individual genes was evaluated using the differential-regulation statistics generated by scTenifoldKnk, including the standardized Z-score and differential-regulation fold change. Multiple-testing correction was performed using the Benjamini–Hochberg method, and genes with an adjusted *P* value < 0.05 were considered significantly perturbed.

Because S100a6 exhibited one of the strongest perturbation signals following virtual Egr1 knockout, the relationship between Egr1 and S100a6 expression was further examined separately in myeloid cells from the day-7 syngeneic and rejection groups using correlation analysis. Significant virtual-KO perturbed genes were subsequently subjected to Gene Ontology (GO) and Kyoto Encyclopedia of Genes and Genomes (KEGG) enrichment analyses using the clusterProfiler R package to characterize the biological processes and signaling pathways potentially associated with the Egr1-centered regulatory network.

### 2.10 Generation and phenotypic maturation of bone marrow-derived dendritic cells

BMDCs were isolated from the femurs and tibias of male Lewis rats and cultured with recombinant rat GM-CSF and IL-4. On day 6, immature BMDCs were harvested and stimulated with lipopolysaccharide (LPS) for 24 h to induce phenotypic maturation. Maturation status was subsequently assessed by evaluating the expression of CD80, CD86, and MHC-II. Detailed isolation and culture protocols are provided in the Supplemental Digital Content (SDC, Materials and Methods).

### 2.11 Lentiviral packaging and BMDC transduction

To establish the *Egr1*-overexpressing BMDC model, primary rat BMDCs were transduced with recombinant lentiviral particles encoding the full-length rat *Egr1* sequence or an empty vector as a negative control (NC). The overexpression efficacy of EGR1 was validated by Western blot analysis prior to subsequent experiments. Where indicated, transduced BMDCs were treated with an EGR1 inhibitor (20 μM, EGR-1-IN-3, MedChemExpress, USA). Detailed protocols for lentiviral vector construction and packaging are provided in the Supplemental Digital Content (SDC, Materials and Methods).

### 2.12 Multiplex immunofluorescence staining

Paraffin-embedded tissue sections were deparaffinized, rehydrated, and subjected to heat-induced antigen retrieval. Following blocking with bovine serum albumin, multiplex IF staining was performed using the tyramide signal amplification (TSA) method. Three independent staining panels were conducted: EGR1 (1:200, ab55160, Abcam, UK) and CD86 (1:200, Epizyme, China); CD4 (1:1000, F12H10, Selleckchem.com) and IL-17A (1:100, ab318150, Abcam, UK); and CD4 (1:1000, F12H10, Selleckchem.com) and FOXP3 (1:300, 85128-6-RR, Proteintech). Appropriate fluorochrome-conjugated secondary antibodies and TSA fluorophores were applied sequentially according to the standard protocols. Nuclei were counterstained with DAPI, and images were captured using a fluorescence microscope. CD4-positive and CD4^+^FOXP3^+^ cells were quantified as the number of positive cells per field. For each animal, cell counts from multiple fields were averaged before statistical analysis.

### 2.13 BMDC-T cell co-culture and CD4^+^ T cell isolation

CD4^+^ T cells were isolated from normal rat spleens using magnetic-activated cell sorting (MACS) with CD4 MicroBeads. To evaluate T cell differentiation, purified CD4^+^ T cells were co-cultured with engineered BMDCs (NC or *Egr1*-overexpressing) at a 1:10 (BMDC: T cell) ratio in 6-well plates for 3 days for subsequent functional assays.

### 2.14 Flow cytometry and T cell proliferation assays

T cell proliferation was assessed using an EdU incorporation assay kit (Beyotime, Shanghai, China). For T cell subset analysis, co-cultured cells were harvested and stained with specific fluorochrome-conjugated antibodies (BioLegend, USA). For intracellular IL-17A detection, the co-cultures were restimulated with PMA and ionomycin in the presence of brefeldin A (BFA, 3 µg/mL) for 4–6 h prior to staining. Following gating on lymphocytes, singlets, and CD4+ T cells, the frequencies of Th17 cells (CD4^+^IL-17A^+^FOXP3^−^) and Tregs (CD4^+^FOXP3^+^IL-17A^−^) were analyzed using FlowJo software.

### 2.15 Statistical analysis

All data are presented as the mean ± standard deviation (SD) or SEM. Statistical analyses for experimental data were performed using GraphPad Prism 10 (San Diego, CA, USA), while bioinformatic evaluations were conducted in R (4.4.1). For comparisons between two independent groups, an unpaired Student’s t-test was used. For comparisons among three or more groups, a one-way or two-way analysis of variance (ANOVA) followed by Tukey’s *post-hoc* test was applied, as appropriate. For nonparametric comparisons, the Mann–Whitney U test or Kruskal–Wallis test followed by Dunn’s *post-hoc* test was used, as appropriate. A *P* value < 0.05 was considered statistically significant.

## 3 Results

### 3.1 Post-transplant histological injury and liver dysfunction in liver grafts

To evaluate the pathophysiological changes following orthotopic liver transplantation, we assessed graft histomorphology, semi-quantitative injury scores, and serum liver enzymes. As shown in **Figure 1A**, the sham group maintained an intact hepatic lobular architecture without obvious inflammatory infiltration or structural damage at all time points.

**Figure 1:**
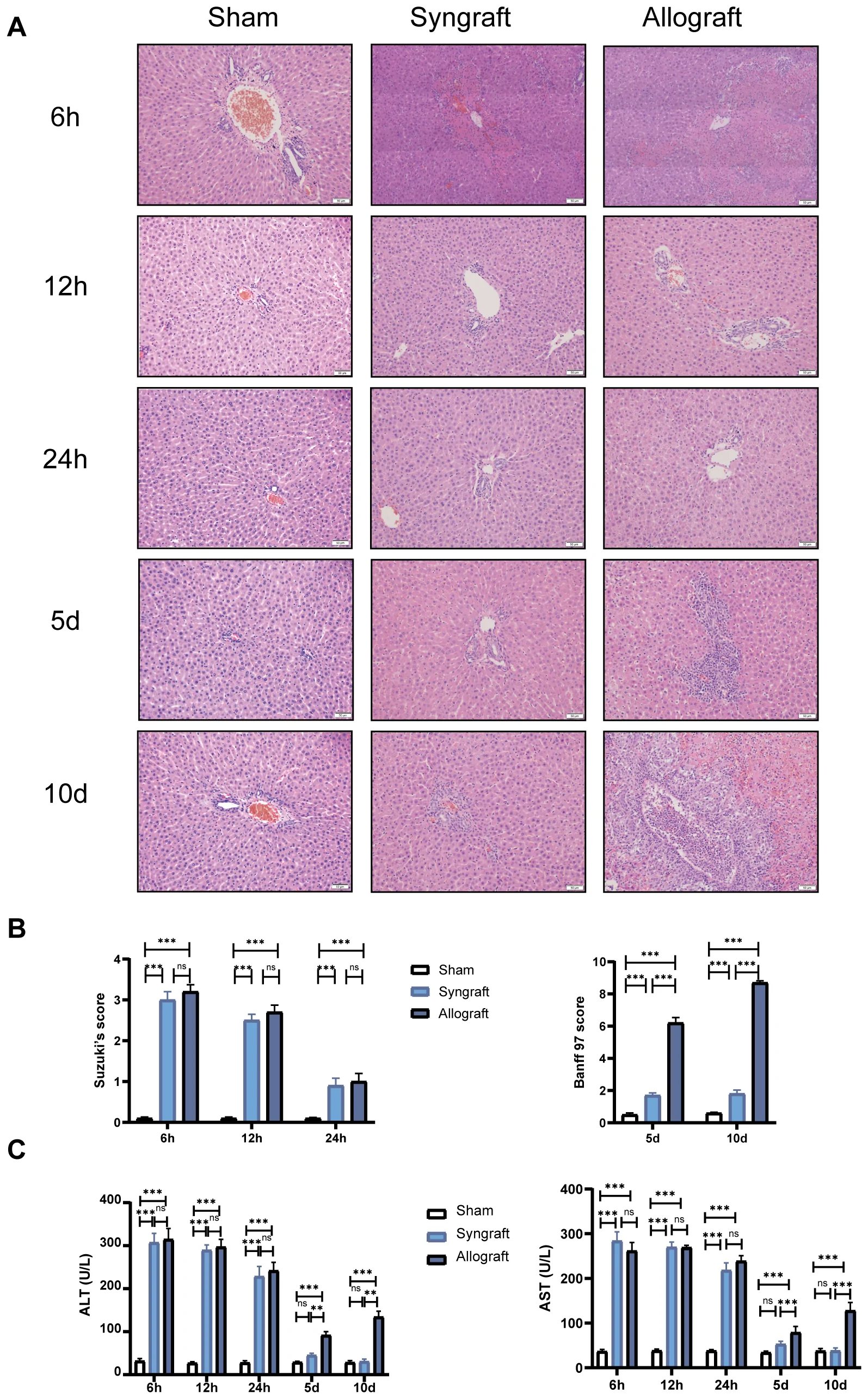
Establishment and validation of the rat liver transplantation model. **(A)** Representative H&E staining images of liver tissues from each group at indicated time points. Scale bars = 50 μm. **(B)** Quantitative analysis of histological damage based on Suzuki’s scores for ischemia-reperfusion injury and RAI scores for acute rejection. **(C)** Serum levels of liver function indicators (ALT and AST) at various time points post-operation. Data are presented as mean ± SD (n = 3 per group). *p < 0.05, **p < 0.01, ***p < 0.001; ns, no significance. ALT, alanine aminotransferase; AST, aspartate aminotransferase; H&E, hematoxylin and eosin.

In the early post-transplantation phase (6–24 h), both the syngraft and allograft groups showed typical histological features of IRI, including sinusoidal congestion and hepatocyte swelling (**Figure 1A**). Accordingly, Suzuki scores increased during the early phase of IRI (**Figure 1B**). Serum ALT and AST levels were markedly elevated and remained high at 24 h, indicating early non-specific hepatocellular damage (**Figure 1C**).

During the late phase (days 5–10), the two groups showed different trends. In the syngraft group, the hepatic architecture gradually recovered, accompanied by decreased Suzuki scores and serum transaminase levels. In contrast, the allograft group developed typical pathological features of AR on days 5–10, characterized by portal inflammatory infiltration, biliary epithelial injury, and endotheliitis (**Figure 1A**; **Supplementary Figure S1**). Consistently, the Banff Rejection Activity Index (RAI) scores increased in the allograft group and peaked on day 10 (**Figure 1B**). Consistent with the histological changes, serum ALT and AST levels in the allograft group were further elevated on day 10 compared to day 5 (**Figure 1C**).

### 3.2 Dynamic changes of cytokine expression in graft tissue and recipient serum after transplantation

Following the characterization of graft injury after transplantation, we further evaluated cytokine expression in local graft tissues and peripheral circulation. RT-qPCR analysis revealed that *Il6* mRNA levels in the allograft liver tissues exhibited a continuous upward trend from days 5 to 10, and were overall higher than those in the syngraft group at the corresponding time points (**Figure 2A**). Additionally, *Il10* mRNA expression significantly increased in the allograft group on days 5–10 (**Figure 2B**), indicating that the rejection phase is accompanied by concurrent changes in immune-regulatory factors.

**Figure 2:**
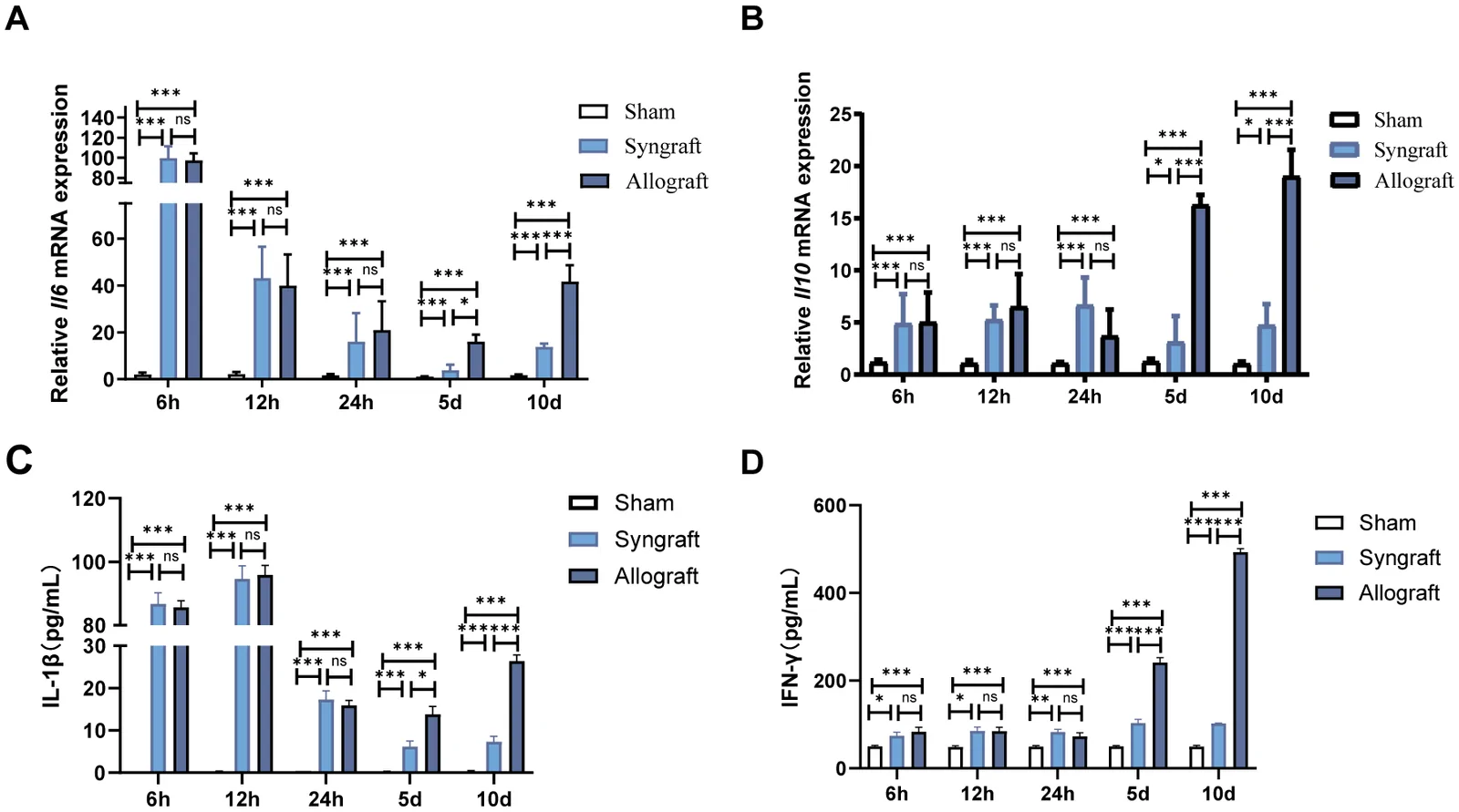
Dynamic changes of cytokine expression in graft tissues and recipient serum post-transplantation. **(A, B)** Relative mRNA expression levels of *IL-6* **(A)** and *IL-10* **(B)** in the liver grafts determined by RT-qPCR. **(C, D)** Serum concentrations of IL-1β **(C)** and IFN-γ **(D)** in the recipients measured by ELISA. Data are presented as mean ± SD (n = 3 per group). *p < 0.05, **p < 0.01, ***p < 0.001; ns, no significance. IL, interleukin; IFN-γ, interferon-gamma; RT-qPCR, reverse transcription quantitative polymerase chain reaction; ELISA, enzyme-linked immunosorbent assay.

In the peripheral circulation, serum ELISA results showed that IL-1β and IFN-γ levels increased during the early post-transplant phase (6–24 h), with similar magnitudes of change observed in both the syngraft and allograft groups (**Figures 2C, D**). During the rejection window (days 5–10), IL-1β and IFN-γ levels in the allograft group further increased and were significantly higher than those in the concurrent syngraft group, with IFN-γ peaking on day 10. These findings indicate enhanced inflammatory responses during acute rejection after allotransplantation.

### 3.3 Single-cell transcriptomic analysis reveals immune remodeling and preferential Egr1 expression in myeloid cells during acute liver allograft rejection

Single-cell RNA-sequencing data from rat liver grafts collected 7 days after transplantation, including three syngeneic and three rejection samples, were analyzed to characterize the cellular landscape and define the cellular context of *Egr1* expression. Quality-control assessment based on the numbers of detected genes and RNA counts showed comparable transcriptomic complexity across the six individual samples following and prior to quality-control filtering, respectively (**Figures 3A, B**). A total of 3,000 highly variable genes were subsequently identified, followed by principal component analysis (PCA) and selection of informative principal components for downstream dimensionality reduction and clustering (**Figures 3C–F**).

**Figure 3:**
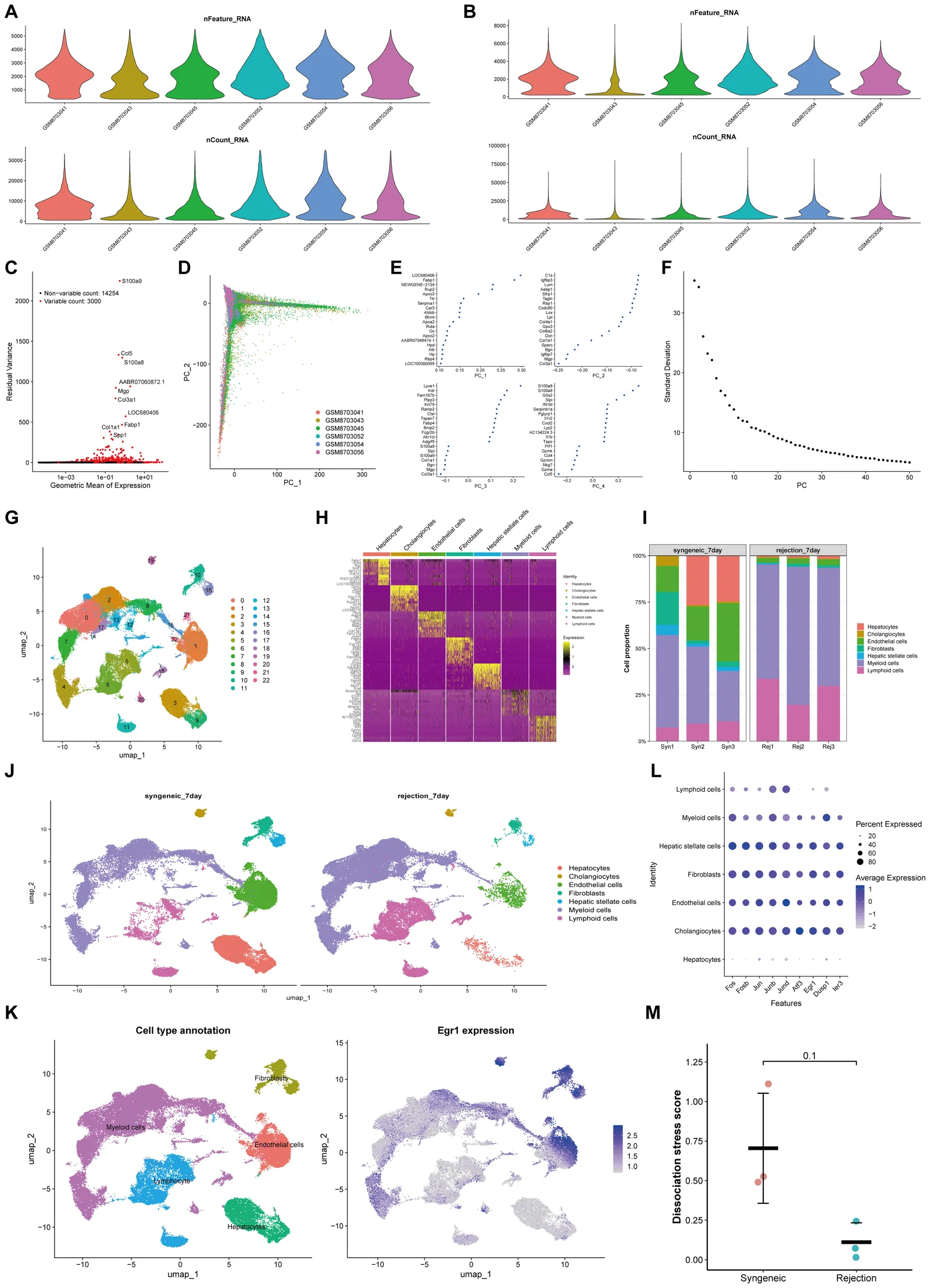
Single-cell transcriptomic analysis of GSE285476 reveals immune-cell remodeling and preferential Egr1 expression in myeloid cells during acute liver allograft rejection. **(A, B)** Violin plots showing the distributions of the number of detected genes (nFeature_RNA) and total transcript counts (nCount_RNA) across the six individual samples following and prior to quality-control filtering, respectively. **(C)** Identification of highly variable genes used for downstream dimensionality reduction; red dots indicate the 3,000 selected variable features. **(D)** Principal component analysis (PCA) showing the distribution of cells from the six individual liver graft samples. **(E)** Genes with the largest contributions to the first four principal components (PC1–PC4). **(F)** Elbow plot showing the standard deviations of the principal components used to guide dimensionality selection. **(G)** UMAP visualization of unsupervised clustering, identifying 23 transcriptionally distinct cell clusters. **(H)** Heatmap showing representative marker-gene expression used for annotation of the major cell populations. **(I)** Relative cellular composition of the syngeneic and rejection groups, showing marked remodeling of the graft cellular landscape during rejection. **(J)** UMAP visualization showing the distribution of the seven major annotated cell populations in the syngeneic and rejection groups. **(K)** UMAP visualization of the seven major annotated cell populations, including hepatocytes, cholangiocytes, endothelial cells, fibroblasts, hepatic stellate cells, myeloid cells, and lymphoid cells (left), together with the distribution of Egr1 expression across the graft cellular landscape (right). **(L)** Dot plot showing the expression of representative stress-responsive and immediate-early genes (Fos, Fosb, Jun, Junb, Jund, Atf3, Egr1, Dusp1, and Ier3) across the major cell populations. Dot size represents the percentage of cells expressing each gene, and color intensity represents average scaled expression. Stress-responsive genes were broadly distributed across multiple cell types, whereas Egr1 showed more prominent expression in myeloid than in lymphoid cells. **(M)** Comparison of dissociation stress scores between the syngeneic and rejection groups. No significant difference was observed between the two groups (P = 0.10).

Unsupervised clustering identified 23 transcriptionally distinct clusters (**Figure 3G**). Based on representative lineage markers, these clusters were annotated into seven major cell populations, including hepatocytes, cholangiocytes, endothelial cells, fibroblasts, hepatic stellate cells, myeloid cells, and lymphoid cells (**Figure 3H**). Comparison of cellular composition revealed marked remodeling of the graft microenvironment in the rejection group, characterized by a predominance of myeloid and lymphoid populations and a corresponding reduction in several parenchymal and stromal populations compared with syngeneic grafts (**Figures 3I, J**).

We next examined the cellular distribution of Egr1. Egr1 was heterogeneously expressed across multiple cell populations rather than being restricted to a single lineage, with relatively prominent expression in several non-parenchymal compartments (**Figure 3K**). Because Egr1 is an immediate-early response gene that may also be sensitive to cell dissociation-associated stress, we further evaluated a panel of immediate-early/stress-associated genes, including Fos, Fosb, Jun, Junb, Jund, Atf3, Egr1, Dusp1, and Ier3. These genes exhibited distinct cell-type-dependent expression patterns, with generally low expression in hepatocytes and relatively higher expression in several non-parenchymal populations (**Figure 3L**). Importantly, the overall dissociation stress score did not differ significantly between the syngeneic and rejection groups (*P* = 0.10; **Figure 3M**), indicating that no systematic increase in dissociation-associated stress was detected in rejection samples. Collectively, these findings define the cellular and pathological context of Egr1 expression and support subsequent cell-type-specific investigation of its regulatory effects.

### 3.4 Temporal expression of EGR1 in liver grafts after transplantation

At the histological level, IHC staining revealed generally weak EGR1 expression in the sham group across all time points. During the early post-transplant phase (6 h), numerous EGR1-positive cells, predominantly exhibiting nuclear staining, were observed in both syngraft and allograft tissues, followed by a signal reduction at 12–24 h (**Figure 4A**). Upon entering the rejection window, distinct divergence emerged: syngrafts displayed predominantly weak positive staining on days 5–10, whereas allografts exhibited markedly enhanced positive signals starting on day 5. This expression was primarily localized in the portal areas and surrounding inflammatory infiltrates, peaking on day 10. Quantification of EGR1-positive cells per field was consistent with these histological observations (**Figure 4A**).

**Figure 4:**
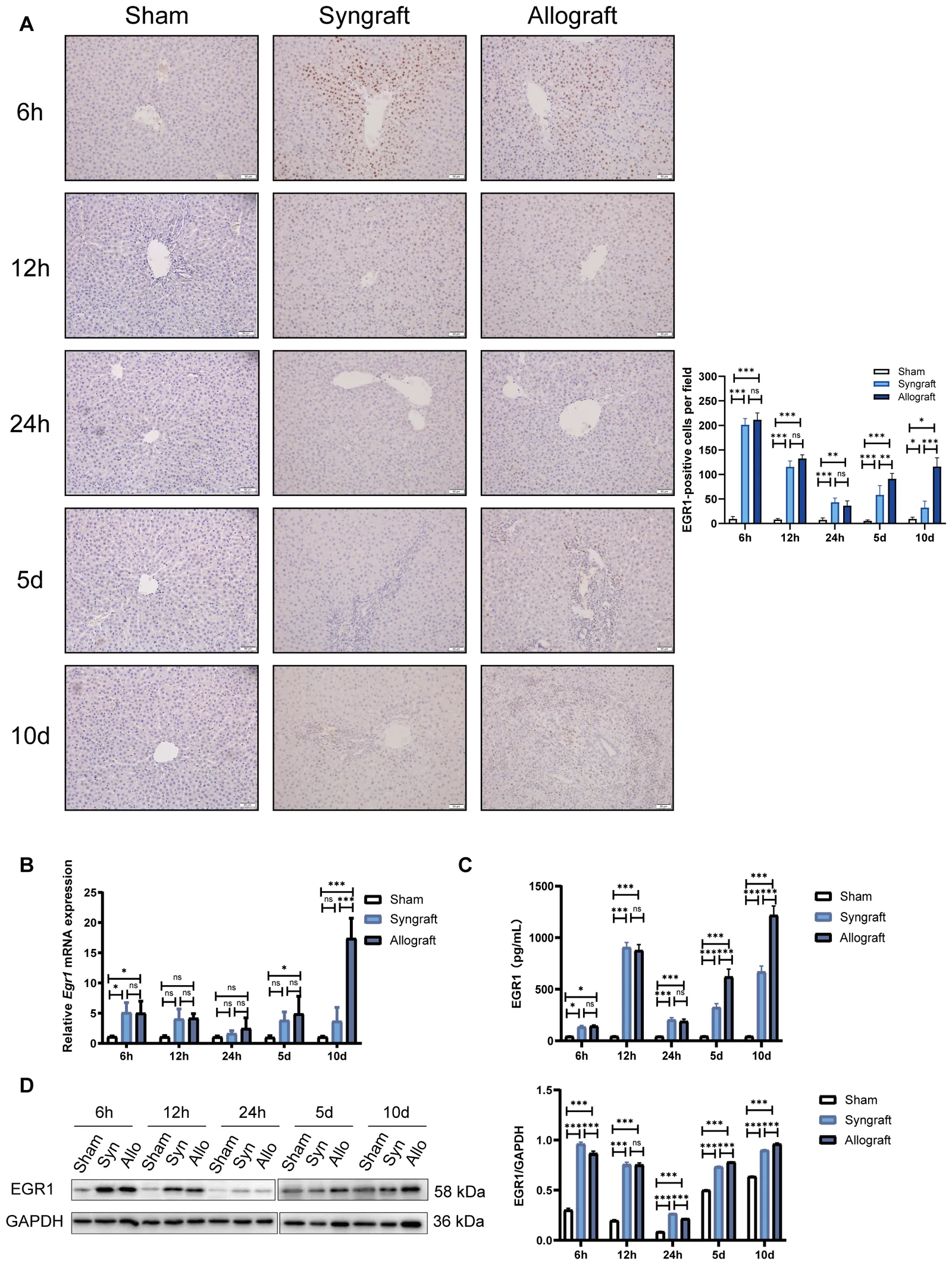
Temporal expression profile of EGR1 in liver grafts post-transplantation. **(A)** Representative immunohistochemistry (IHC) images and quantification of EGR1-positive cells per field in liver tissues from the Sham, Syngraft, and Allograft groups at 6 h, 12 h, 24 h, 5 d, and 10 d post-operation. Quantification was performed by counting EGR1-positive cells per field. Scale bars = 50 μm. **(B)** Relative mRNA expression levels of *Egr1* in liver grafts determined by RT-qPCR. **(C)** EGR1 protein concentrations in the respective groups. **(D)** Representative Western blot images (left) and densitometric quantification (right) of EGR1 protein expression in liver grafts at indicated time points. Data are presented as mean ± SD (n = 3 per group). *p < 0.05, **p < 0.01, ***p < 0.001; ns, no significance. Allo, allograft; GAPDH, glyceraldehyde-3-phosphate dehydrogenase; IHC, immunohistochemistry; RT-qPCR, reverse transcription quantitative polymerase chain reaction; Syn, syngraft.

Molecular assays further supported these histological observations. RT-qPCR showed an early increase in *Egr1* mRNA in both transplant groups, whereas by day 10, *Egr1* expression was markedly higher in allografts than in syngrafts and sham controls (**Figure 4B**). EGR1 protein levels also increased during the early phase and declined after 24 h, followed by a secondary increase in allografts during days 5-10 (**Figure 4C**). Western blot analysis and densitometric quantification showed a similar temporal pattern (**Figure 4D**).

Taken together, these findings demonstrate a biphasic pattern of EGR1 expression after transplantation, characterized by an early increase in both transplant groups and a secondary increase in allografts during acute rejection. Together with the day 7 scRNA-seq findings, these results supported further investigation of myeloid EGR1.

### 3.5 Virtual knockout analysis of Egr1 in graft myeloid cells

To explore the potential regulatory role of Egr1 in graft myeloid cells, we performed a virtual-KO analysis using the scTenifoldKnk framework. scTenifoldKnk constructs a single-cell gene regulatory network (scGRN), generates a pseudo-KO network by virtually removing the regulatory influence of the target gene, and compares the original and perturbed networks using manifold alignment to DR genes, hereafter referred to as virtual-KO perturbed genes.

Virtual knockout of Egr1 resulted in significant network perturbation in a relatively small subset of genes, with approximately 0.2% of the analyzed genes meeting the significance criterion (**Figure 5A**). Ranking genes according to the differential-regulation output identified S100a6 and S100a4 as the most strongly perturbed genes, followed by MGC105649, Anxa2, and Lsp1 (**Figure 5B**). Consistently, visualization of the differential-regulation statistics showed that S100a6 and S100a4 exhibited the most prominent perturbation signals, with MGC105649 and Anxa2 also showing significant changes in network regulation following virtual Egr1 knockout (**Figure 5C**). These results suggest that virtual loss of Egr1 preferentially perturbs a discrete set of genes within the myeloid regulatory network rather than inducing widespread nonspecific network changes.

**Figure 5:**
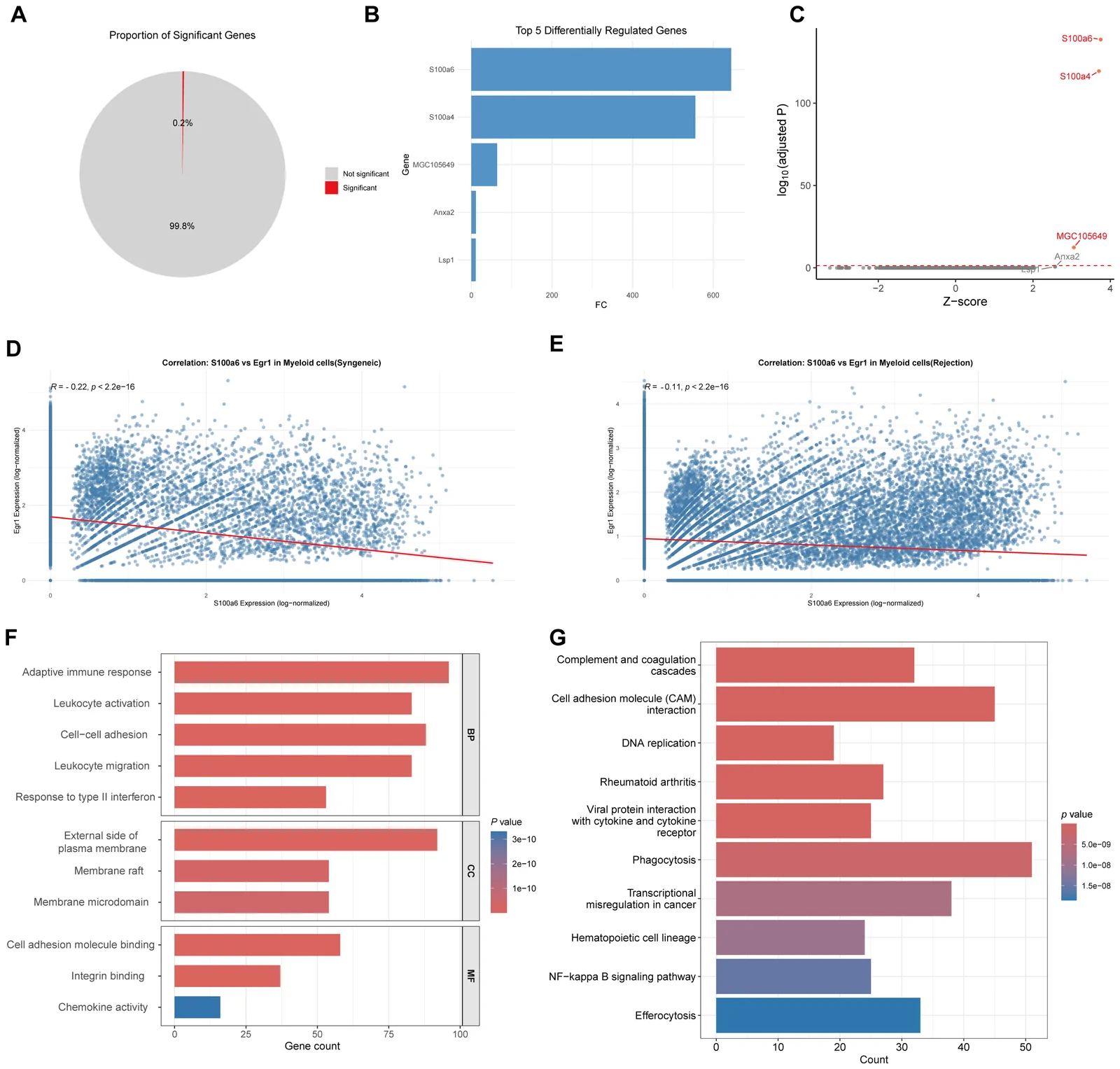
Virtual knockout analysis of Egr1 reveals immune-related regulatory perturbations in graft myeloid cells. **(A)** Proportion of significant and non-significant differentially regulated genes following virtual Egr1 knockout. Approximately 0.2% of the analyzed genes met the significance criterion. **(B)** Top five virtual-KO perturbed genes ranked according to the differential-regulation fold change (FC) relative to the expected network-perturbation level. S100a6, S100a4, MGC105649, Anxa2, and Lsp1 showed the strongest perturbation signals. **(C)** Differential-regulation plot showing the Z-score and −log10-adjusted *P* value for individual genes following virtual Egr1 knockout. Genes with prominent perturbation signals are labeled. **(D, E)** Correlation between S100a6 and Egr1 expression in myeloid cells from the day-7 syngeneic **(D)** and rejection **(E)** groups. S100a6 was weakly negatively correlated with Egr1 in both groups (syngeneic: *R* = −0.22, *P* < 2.2 × 10^-^¹^6^; rejection: *R* = −0.11, *P* < 2.2 × 10^-^¹^6^). Each point represents an individual myeloid cell, and the red line indicates the fitted trend. **(F)** Gene Ontology enrichment analysis of significant virtual-KO perturbed genes, showing representative Biological Process (BP), Cellular Component (CC), and Molecular Function (MF) terms. **(G)** Kyoto Encyclopedia of Genes and Genomes (KEGG) enrichment analysis of significant virtual-KO perturbed genes, highlighting pathways related to complement and coagulation cascades, cell adhesion, phagocytosis, hematopoietic cell lineage, NF-κB signaling, and efferocytosis. DR, differential regulation; FC, fold change relative to the expected differential-regulation distance; GO, Gene Ontology; KEGG, Kyoto Encyclopedia of Genes and Genomes.

Because S100a6 exhibited the strongest perturbation signal, we further examined its transcriptional relationship with Egr1 in myeloid cells from the day-7 syngeneic and rejection groups. S100a6 expression was weakly but significantly negatively correlated with Egr1 expression in both conditions, with a stronger association in the syngeneic group (*R* = −0.22, *P* < 2.2 × 10^-^¹^6^; **Figure 5D**) than in the rejection group (*R* = −0.11, *P* < 2.2 × 10^-^¹^6^; **Figure 5E**). Given the modest correlation coefficients, these findings support a network-level association between Egr1 and S100a6 but do not establish direct transcriptional regulation.

Functional enrichment analysis of the significant virtual-KO perturbed genes further suggested that the Egr1-associated regulatory network was closely related to immune and myeloid-cell functions. Gene Ontology analysis identified enrichment of biological processes including adaptive immune response, leukocyte activation, cell-cell adhesion, leukocyte migration, and response to type II interferon. Enriched cellular-component terms included the external side of the plasma membrane, membrane raft, and membrane microdomain, whereas molecular-function terms included cell adhesion molecule binding, integrin binding, and chemokine activity (**Figure 5F**). KEGG analysis further highlighted complement and coagulation cascades, cell adhesion molecule interactions, phagocytosis, hematopoietic cell lineage, NF-κB signaling, and efferocytosis, among other immune-related pathways (**Figure 5G**). Collectively, the virtual-KO analysis suggests that Egr1 is associated with regulatory programs related to immune responses, adhesion, migration, and phagocytic functions in graft myeloid cells.

### 3.6 Enhanced Th17-associated immune responses in liver allografts during acute rejection

To further characterize the immune response during acute rejection, we examined myeloid activation and T cell-associated markers in liver grafts. Histological analysis showed increased CD86 immunoreactivity in allografts during the rejection phase, with the CD86-positive area reaching its highest level on day 10 (**Figure 6A**). Multiplex immunofluorescence showed partial colocalization of EGR1 and CD86 signals in periportal areas (**Figure 6B**), suggesting an association between EGR1 expression and local APCs activation during acute rejection.

**Figure 6:**
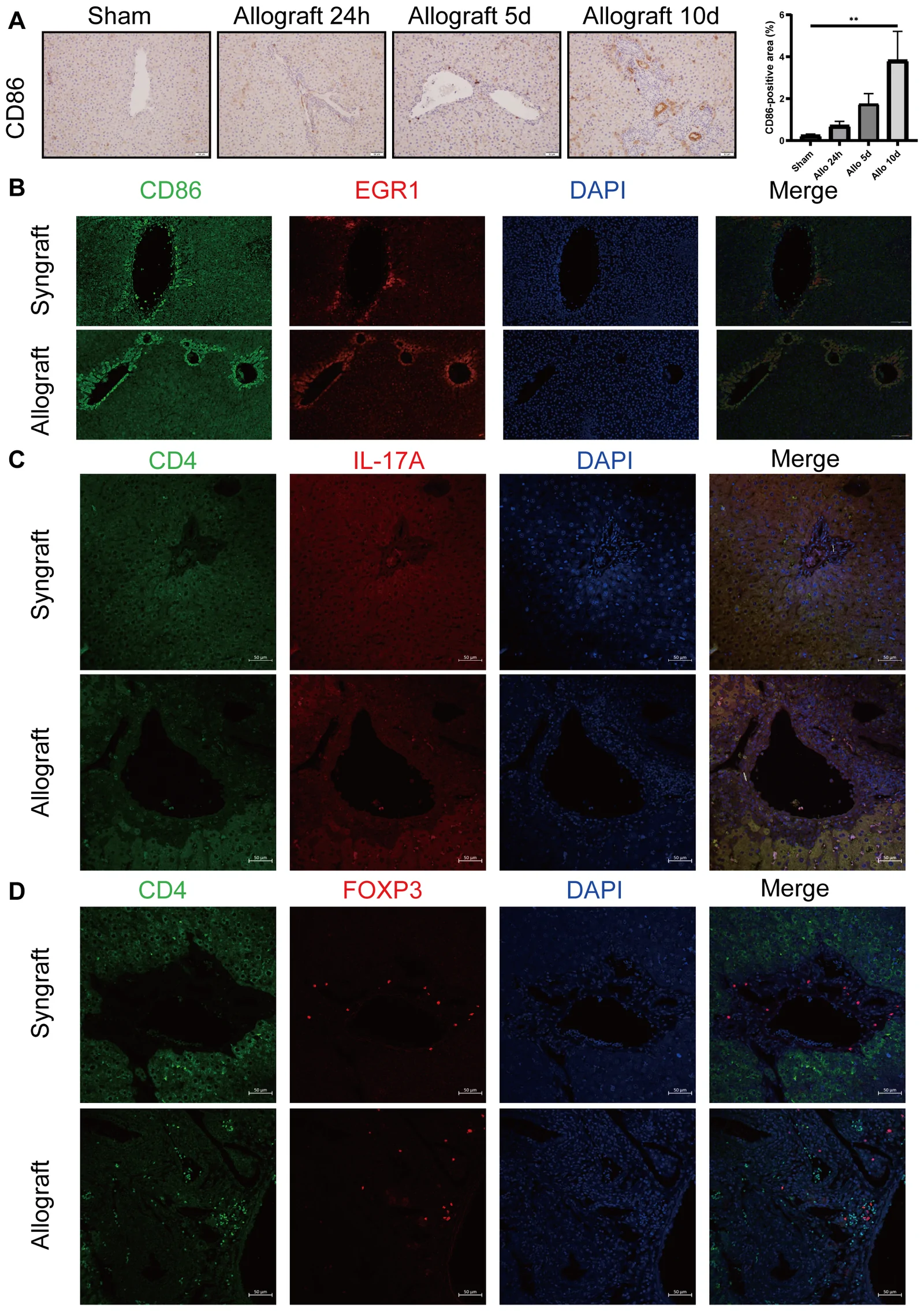
EGR1-expressing myeloid cells and T cell-associated immune responses in liver allografts during acute rejection. **(A)** Representative immunohistochemistry (IHC) images and quantitative analysis of CD86-positive area in liver grafts from the Sham and Allograft groups at indicated time points after transplantation. **(B)** Representative multiplex immunofluorescence (mIF) staining showing CD86 (green) and EGR1 (red) expression in liver grafts. DAPI (blue) was used for nuclear counterstaining. **(C)** Representative mIF staining of CD4 (green) and IL-17A (red) in syngraft and allograft liver tissues. **(D)** Representative mIF staining of CD4 (green) and FOXP3 (red) in syngraft and allograft liver tissues. Scale bars = 50μm. EGR1, early growth response 1; IHC, immunohistochemistry; mIF, multiplex immunofluorescence; DAPI, 4’,6-diamidino-2-phenylindole; FOXP3, forkhead box P3.

Given that activated myeloid APCs regulate T-cell polarization (22, 23), we further evaluated the downstream T-cell responses. IL-17A immunohistochemical staining was more prominent in allografts than in syngrafts at day 10, although the quantitative difference did not reach statistical significance (**Supplementary Figure S2A**). Multiplex immunofluorescence further showed more prominent CD4/IL-17A signals in allografts (**Figure 6C**), consistent with enhanced Th17-associated infiltration during acute rejection. CD4/FOXP3 staining was also examined to assess Treg-associated responses (**Figure 6D**), with quantitative analyses of CD4-positive and CD4^+^FOXP3^+^ cells shown in **Supplementary Figures S2B, C**. These *in vivo* histological findings prompted subsequent investigation of myeloid EGR1-mediated T-cell responses *in vitro*.

### 3.7 EGR1 overexpression in BMDCs enhances CD4^+^ T-cell proliferation and skews Th17/Treg balance *in vitro*

To assess the effects of EGR1 on DC function and T-cell polarization, we generated rat BMDCs. Flow cytometry confirmed that LPS stimulation upregulated the maturation markers CD80, CD86, and MHC-II (**Figure 7A**). BMDCs were then transduced with an *Egr1* overexpression (OE) lentiviral vector and treated with an EGR1 inhibitor. Western blot analysis verified EGR1 upregulation in the OE group and its suppression by the inhibitor (**Figure 7B**).

**Figure 7:**
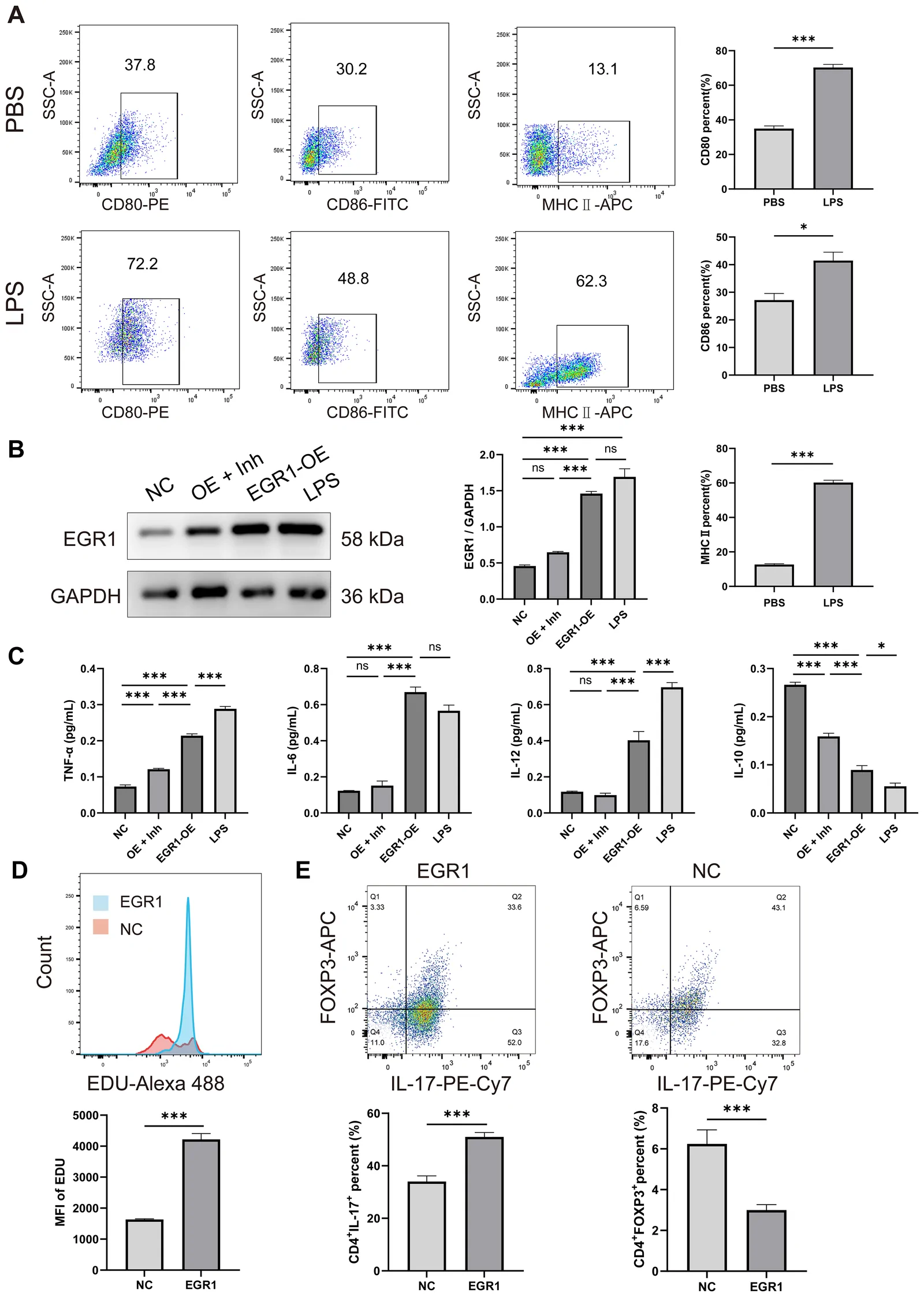
EGR1-overexpressing BMDCs promote CD4^+^ T-cell proliferation and alter Th17/Treg differentiation *in vitro*. **(A)** Flow cytometry analysis and quantification of the surface maturation markers CD80, CD86, and MHC-II on bone marrow-derived dendritic cells (BMDCs) treated with PBS or LPS. **(B)** Western blot analysis (left) and quantification (right) verifying EGR1 overexpression (EGR1-OE) in BMDCs and its suppression by an EGR1 inhibitor (OE + Inh). **(C)** ELISA quantification of pro-inflammatory cytokines (TNF-α, IL-6, IL-12) and the anti-inflammatory cytokine (IL-10) in the BMDC culture supernatants. **(D)** CD4^+^T cell proliferation assessed by EdU incorporation assay following co-culture with negative control (NC) or EGR1-overexpressing BMDCs. **(E)** Flow cytometry analysis of intracellular cytokines and transcription factors in co-cultured CD4^+^ T cells. Representative dot plots (left) and statistical quantification (right) showing the proportions of pro-inflammatory Th17 cells (CD4^+^IL-17A^+^FOXP3^-^) and immunosuppressive Tregs (CD4^+^FOXP3^+^IL-17A^-^) in the NC and EGR1 groups. The gating strategy is shown in **Supplementary Figure S3**. Data are presented as mean ± SEM of three independent experiments. *p < 0.05, ***p < 0.001. EGR1, early growth response 1; BMDC, bone marrow-derived dendritic cell; LPS, lipopolysaccharide; OE, overexpression; Inh, inhibitor; ELISA, enzyme-linked immunosorbent assay; EdU, 5-ethynyl-2’-deoxyuridine; MFI, mean fluorescence intensity; Treg, regulatory T cell; Th17, T helper 17 cell; FOXP3, forkhead box P3; IL-17, interleukin-17.

ELISA analysis of the culture supernatants showed that EGR1 overexpression increased the levels of IL-6, IL-12, TNF-α and decreased IL-10 (**Figure 7C**). Treatment with the EGR1 inhibitor reversed these cytokine changes.

In BMDC-CD4^+^ T cell co-culture assays, EdU incorporation (**Figure 7D**) indicated that EGR1-overexpressing BMDCs promoted CD4^+^ T-cell proliferation. Furthermore, flow cytometry of the co-cultured T cells revealed an increased proportion of Th17 cells (CD4^+^IL-17A^+^FOXP3^-^) and a decreased proportion of Tregs (CD4^+^FOXP3^+^IL-17A^-^) in the OE group (**Figure 7E**). Collectively, these *in vitro* results indicate that EGR1 overexpression in BMDCs enhances CD4^+^ T-cell proliferation and shifts Th17/Treg differentiation under the co-culture conditions.

## 4 Discussion

Despite the widespread use of immunosuppressants, the long-term survival of liver allografts remains limited by IRI and AR (3, 4). In this study, we combined a rat orthotopic liver transplantation model with single-cell transcriptomics, computational gene perturbation, histological analysis, and *in vitro* functional experiments to investigate the temporal and cellular characteristics of EGR1 during liver transplantation. EGR1 exhibited a biphasic expression pattern, with transient induction during early IRI followed by renewed upregulation during established AR. Single-cell analysis further defined the cellular context of Egr1 during rejection, whereas virtual perturbation in graft-derived myeloid cells implicated EGR1-associated immune regulatory programs. Histological and BMDC–T-cell experiments subsequently supported a potential role for myeloid EGR1 in modulating inflammatory responses and downstream CD4^+^ T-cell differentiation.

First, our dynamic evaluation of histology and serum liver enzymes temporally distinguished IRI from AR in the rat model. At 6–24 hours post-transplantation, both syngraft and allograft groups exhibited comparable injury severity. This early similarity is consistent with IRI being largely associated with sterile inflammatory responses rather than alloantigen-specific immunity (3, 5). Conversely, at 5–10 days, only the allograft group developed histological features of AR along with a secondary rebound in liver enzymes, consistent with the typical timeline of acute rejection in rats (24, 25). As an immediate-early response transcription factor, EGR1 is rapidly induced by stimuli such as hypoxia, inflammatory mediators, and tissue injury (26). Accordingly, its transient elevation in both graft groups during the early postoperative period likely reflects a stress-associated response to IRI. During AR, however, EGR1 was upregulated again specifically in allografts and was prominently detected in portal inflammatory infiltrates. This biphasic pattern suggests that EGR1 activation occurs in distinct pathological contexts during transplantation: an early response associated with ischemic injury and a later response accompanying alloimmune inflammation. These findings distinguish stage-associated EGR1 responses without implying that its early induction during IRI directly causes subsequent rejection.

Previous studies in cardiac and lung transplant models have associated overall EGR1 upregulation with allograft rejection (10, 13). Consistent with this, we observed a transient elevation of EGR1 in both graft groups during the IRI phase, consistent with an early stress-associated response. However, during the AR phase, EGR1 was significantly upregulated again in the allografts, with prominent EGR1-positive signals in portal inflammatory infiltrates. This biphasic pattern suggests that early EGR1 induction is associated with IRI-related stress, whereas the secondary increase accompanies alloimmune rejection (8). Thus, examining both phases helped distinguish these stage-associated EGR1 responses without implying a causal relationship between IRI and AR.

Single-cell transcriptomic analysis provided more precise insights into the *Egr1*-expressing cell populations. In contrast to the global upregulation of EGR1 observed at the bulk tissue level, day 7 scRNA-seq showed that Egr1 was expressed across multiple graft cell populations but was preferentially enriched in myeloid cells within the immune compartment. Notably, *Egr1* was also part of a broader stress response program (27). This preferential myeloid expression is congruent with recent genomic studies identifying EGR1 as a master transcriptional regulator of macrophage functional networks (28), and is consistent with the established importance of myeloid cells in allograft rejection (29). Thus, the single-cell data define the cellular distribution of *Egr1* during established rejection but do not establish myeloid-specific temporal dynamics.

The stress-responsive nature of EGR1 is particularly relevant to the interpretation of scRNA-seq data. Immediate-early genes such as Egr1, Fos, Jun, and Atf3 can be induced during tissue dissociation, potentially introducing transcriptional artifacts. In our dataset, these genes displayed cell-type-dependent rather than uniformly elevated expression, and the overall dissociation-stress score did not differ significantly between syngeneic and rejection samples. Although this cannot completely exclude an effect of tissue processing on individual genes, it argues against a systematic increase in dissociation-associated stress in the rejection group. The observed Egr1 pattern should therefore be interpreted together with the histological and functional evidence rather than attributed solely to tissue processing. Within this context, we further explored the potential regulatory functions associated with Egr1 in graft myeloid cells.

To explore the potential downstream functions of myeloid EGR1, we applied virtual knockout analysis to day 7 graft myeloid cells. The analysis identified a discrete set of Egr1-associated regulatory perturbations, with enrichment in immune responses, leukocyte activation and migration, cell adhesion, phagocytosis, and NF-κB signaling. These findings are consistent with previous evidence linking EGR1 to inflammatory regulation in activated macrophages (30). Given that IL-1β and IL-6 are established regulators of Th17 differentiation (31, 32), these immune-related regulatory programs may provide a biological context for the subsequent effects of myeloid EGR1 on T-cell responses. However, the virtual knockout analysis does not establish these cytokines as direct downstream mediators of EGR1. Virtual knockout reflects network perturbation rather than experimental gene deletion, and its interpretation is specific to day-7 graft myeloid cells.

Acute cellular rejection is fundamentally driven by alloreactive T cells, which require activation by APCs within the allograft microenvironment (19, 23). Histologically, we observed that EGR1 protein expression partially colocalized with CD86 signals in the periportal areas. This anatomical localization is critical, as portal tracts serve as the primary sites for immune cell infiltration and initial antigen presentation during liver allograft rejection (6), suggesting that the spatial distribution of EGR1 is associated with local APC activation. *In vivo* histological analysis showed more prominent Th17 associated staining in allografts, together with CD4/FOXP3 signals, accompanied by increased graft *Il6* expression and serum IL-1β levels. These observations align with literature showing that Th17 differentiation depends on these cytokines and APC co-stimulation (31, 32). *In vitro* assays demonstrated that EGR1 overexpression in BMDCs increased pro-inflammatory cytokine secretion, enhanced CD4^+^ T-cell proliferation, and shifted Th17/Treg differentiation. Collectively, these findings suggest that myeloid EGR1 may modulate the local inflammatory environment and influence T-cell polarization during acute rejection.

To assess the functional effects of EGR1, we employed a BMDC and CD4^+^ T-cell co-culture system for functional validation. The results demonstrated that EGR1 overexpression in BMDCs enhanced CD4^+^ T-cell proliferation, significantly promoted Th17 differentiation, and concurrently suppressed Treg differentiation. This *in vitro* finding is consistent with recent reports demonstrating that EGR1 regulates T-cell differentiation in other inflammatory contexts (28, 33, 34). Notably, whether EGR1 directly regulates the transcription of classical DC maturation markers (e.g., CD80, CD86, and MHC-II) remains to be elucidated. Deciphering these precise molecular interactions will be a primary focus of our subsequent mechanistic studies.

Interestingly, although EGR1 has been reported to suppress inflammatory responses in certain contexts (35) or promote tissue repair in drug-induced liver injury (36), our findings support a pro-inflammatory role for EGR1 in the alloreactive environment of liver rejection. This suggests that EGR1 functions are highly context-dependent. Crucially, inhibiting EGR1 reversed the pro-inflammatory secretory phenotypes of BMDCs *in vitro*, supporting its potential as a target for immune modulation during acute rejection.

From a translational perspective, the preferential enrichment of EGR1 in graft myeloid cells during acute rejection suggests a potential basis for targeted immunomodulation. *In vitro* EGR1 inhibition reversed the pro-inflammatory cytokine profile of BMDCs, supporting the potential of EGR1 as an immunomodulatory target. If validated *in vivo*, targeting EGR1 may provide an alternative approach to broad T-cell suppression and its associated adverse effects (37, 38).

Several limitations should be acknowledged. First, the transplantation experiments included a relatively small number of animals, which may limit statistical power and the robustness of intergroup comparisons. In addition, the transplantation experiments were performed in rats, whereas EGR1 regulatory networks may vary across species and experimental models. Second, scRNA-seq was restricted to day 7 and therefore represents the cellular state of established rejection rather than the temporal evolution of individual Egr1-expressing populations from IRI to AR. Third, although dissociation-stress scores did not differ significantly between groups, an influence of tissue processing on individual stress-responsive genes cannot be completely excluded. Fourth, virtual knockout represents network-based prediction rather than experimental gene deletion and cannot establish direct transcriptional causality. Finally, definitive *in vivo* evidence will require myeloid-specific Egr1 loss-of-function models and further identification of the downstream mechanisms linking EGR1 to APC function and Th17/Treg responses. Validation in human liver transplant samples will also be required to establish clinical relevance.

Collectively, our study identifies myeloid EGR1 as a potential regulator of Th17/Treg responses during acute liver allograft rejection. These findings suggest that EGR1 may connect myeloid APC activation with downstream T-cell differentiation, providing a rationale for further evaluation of EGR1-targeted immunomodulation.

## Data Availability

The datasets presented in this study can be found in online repositories. The names of the repository/repositories and accession number(s) can be found in the article/**Supplementary Material**.

## Glossary

APC: Antigen-presenting cell
AR: Acute rejection
BMDCs: Bone marrow-derived dendritic cells
EGR1: Early growth response 1
IF: Immunofluorescence
IHC: Immunohistochemistry
IRI: Ischemia-reperfusion injury
LPS: Lipopolysaccharide
MACS: Magnetic-activated cell sorting
MFI: Mean fluorescence intensity
RAI: Rejection Activity Index
ROLT: Rat orthotopic liver transplantation
scRNA-seq: Single-cell RNA sequencing
Th17: T helper 17 cells
Tregs: Regulatory T cells
