# Supplementary material for: Myeloid EGR1 contributes to Th17/Treg imbalance during acute liver allograft rejection in a rat model
Source: Front Immunol. 2026 Sep 21;17:1901189. doi: 10.3389/fimmu.2026.1901189 (PMC13634955; doi:10.3389/fimmu.2026.1901189)
Supplement: Supplementary file 1 [file SupplementaryFile1.docx]

**SDC, Materials and Methods**

**Table S1. Primer sequences used for RT-qPCR**

| Gene | NCBI RefSeq accession No. | Forward Primer (5′→3′) | Reverse Primer (5′→3′) |
| --- | --- | --- | --- |
| *Egr1* | NM_012551.3 | AACAACCCTACGAGCACCTG | AAAGGGGTTCAGGCCACAAA |
| *Il-6* | NM_012589.2 | AGCGATGATGCACTGTCAGA | TAGCACACTAGGTTTGCCGA |
| *Il-10* | NM_012854.2 | TTGAACCACCCGGCATCTAC | CCAAGGAGTTGCTCCCGTTA |
| *18S rRNA* | NR_046237.3 | GCCATGCATGTCTAAGTACGC | CCGTCGGCATGTATTAGCTC |

**SDC, Generation and phenotypic maturation of BMDCs**

BMDCs were isolated and generated from the bone marrow of the femurs and tibias of male Lewis rats. Briefly, the bone marrow cavities were flushed with sterile PBS, and the resulting cell suspension was filtered through a 70-μm cell strainer and centrifuged. The cell pellets were resuspended and cultured in RPMI 1640 medium supplemented with 10% fetal bovine serum (FBS), 1% penicillin-streptomycin, recombinant rat GM-CSF (20 ng/mL; PeproTech, USA), and recombinant rat IL-4 (10 ng/mL; PeproTech, USA). The culture medium was half-replaced every 2 days with fresh cytokine-supplemented medium. On day 6, the non-adherent and loosely adherent cells were harvested as immature BMDCs. To induce phenotypic maturation, these cells were stimulated with lipopolysaccharide (LPS; 1 μg/mL; Sigma-Aldrich, USA) for an additional 24 h.

**SDC, Lentiviral vector construction and packaging**

To establish the *Egr1*-overexpressing DC model, a recombinant lentiviral vector was constructed by cloning the full-length coding sequence of rat *Egr1* into the multiple cloning site of the pCDH-CMV-MCS-3xFlag-EF1-copGFP-T2A-Puro plasmid (ZOLGEEN, China). For lentivirus production, the *Egr1* overexpression plasmid or the empty vector (Negative Control, NC) was co-transfected with appropriate lentiviral packaging plasmids into HEK-293T cells using the Lipofectamine 3000 reagent (Invitrogen, Carlsbad, CA, USA) according to the manufacturer's protocols. Viral supernatants were harvested at 48 h and 72 h post-transfection, centrifuged, and filtered. Subsequently, the primary rat BMDCs were transduced with the collected lentiviral particles.

**SDC, Supplementary Figures S1.** **Representative high-magnification histopathological features of acute rejection in rat liver allografts.**

**Figure S1**. (A) Representative H&E images showing biliary epithelial injury in an allograft at day 10 after transplantation. (B) Representative H&E image showing venous endotheliitis in an allograft at day 10 after transplantation. Scale bars = 20 μm.

**SDC, Supplementary Figure S2. Quantitative analysis of T cell-associated markers in liver grafts during acute rejection.**

**Figure S2.** (A) Representative immunohistochemistry (IHC) images showing IL-17A staining and quantitative analysis of IL-17A-positive area in syngraft and allograft liver tissues at day 10 after transplantation. (B) Quantitative analysis of CD4-positive cells in liver grafts. (C) Quantitative analysis of CD4⁺FOXP3⁺ cells in liver grafts. Data are presented as mean ± SD (n = 3 per group). Scale bars = 100 μm (A)

**SDC, Supplementary Figure S3.** **Flow cytometry gating strategy for CD4⁺ T-cell analysis.**

Representative gating strategy showing the identification of lymphocyte populations, single cells, CD4⁺ T cells, and subsequent analysis of IL-17A and FOXP3 expression in CD4⁺ T cells.
